# Sequencing, Characterization, and Comparative Analyses of the Plastome of *Caragana rosea* var. *rosea*

**DOI:** 10.3390/ijms19051419

**Published:** 2018-05-09

**Authors:** Mei Jiang, Haimei Chen, Shuaibing He, Liqiang Wang, Amanda Juan Chen, Chang Liu

**Affiliations:** Key Laboratory of Bioactive Substances and Resource Utilization of Chinese Herbal Medicine from Ministry of Education, Institute of Medicinal Plant Development, Chinese Academy of Medical Sciences, Peking Union Medical College, Beijing 100193, China; mjiang0502@163.com (M.J.); hmchen@implad.ac.cn (H.C.); wenyuxuan2530@163.com (S.H.); lys832000@163.com (L.W.); amanda_j_chen@163.com (A.J.C.)

**Keywords:** *Caragana*, *Caragana rosea* var. *rosea*, plastome, comparative genomics, molecular markers

## Abstract

To exploit the drought-resistant *Caragana* species, we performed a comparative study of the plastomes from four species: *Caragana rosea*, *C. microphylla*, *C. kozlowii*, and *C. Korshinskii*. The complete plastome sequence of the *C. rosea* was obtained using the next generation DNA sequencing technology. The genome is a circular structure of 133,122 bases and it lacks inverted repeat. It contains 111 unique genes, including 76 protein-coding, 30 tRNA, and four rRNA genes. Repeat analyses obtained 239, 244, 258, and 246 simple sequence repeats in *C. rosea*, *C. microphylla*, *C. kozlowii*, and *C. korshinskii*, respectively. Analyses of sequence divergence found two intergenic regions: *trnI*-*CAU*-*ycf2* and *trnN*-*GUU*-*ycf1*, exhibiting a high degree of variations. Phylogenetic analyses showed that the four *Caragana* species belong to a monophyletic clade. Analyses of Ka/Ks ratios revealed that five genes: *rpl16*, *rpl20*, *rps11*, *rps7,* and *ycf1* and several sites having undergone strong positive selection in the *Caragana* branch. The results lay the foundation for the development of molecular markers and the understanding of the evolutionary process for drought-resistant characteristics.

## 1. Introduction

The genus *Caragana* has more than 100 species and it belongs to the family of Leguminosae. The plants mainly grow in arid and semi-arid areas of Asia and Europe. Plants from this genus are well-known in resisting drought, barren, cold, and heat, and have a strong adaptability to the sand environment to prevent wind and fixate sand [1]. Sixty two species of *Caragana* are distributed in China [2], most of them can afforest barren hills and preserve water and soil. The distributions of several *Caragana* species have been well-studied in China. *C. microphylla* and *C. korshinskii* are distributed in Northeast China, North China, and Northwest China [3]. *C. microphylla* is adapted to the typical steppe zone, forest steppe zone, and deciduous broad-leaved forest steppe zone of the Mongolian plateau. *C. korshinskii* is suitable for the fixed and semi fixed sand land in the steppe desert and the typical desert belt zone. *C kozlowii* origins in the Lancang River and Tibet, and was mostly found in riverside with 3600–4000 m altitude [4]. *C rosea* is mainly from Northeast China, North China, East China, Henan, and southern Gansu Province, growing in slopes and valleys [5]. In addition to its drought adaptability, its medicinal value, such as strengthening the spleen and tonifying the kidney, was also well known [1]. Furthermore, it was shown that chemical constituents in *C. rosea* have anti-HIV activities [6]. Until now, the plastome of *C. korshinskii*, *C. microphylla,* and *C. kozlowii* were reported, while the plastome of *C. rosea* has not been studied.

Plants live in constantly changing environments that impose many biotic stress, such as pathogen infection and herbivore attack and abiotic stress, such as drought, heat, cold, nutrient deficiency, and excess of salt or toxic metals. Through the past years, many abiotic stress signaling and response pathways in plants have been discovered, with the core pathways involve protein kinases related to the yeast SNF1 and mammalian AMPK [7]. There is also research from the evolution of chloroplast genes adapted to contrasting habitats. For example, the *Cardamine resedifolia* plastid gene has undergone a more aggressive positive selection than *Cardamine impatiens,* which is located at lower elevations, which is why it is more adapted to the plateau environment [8,9]. For *Caragana* species, dozens of studies have been carried out on the morphological changes, such as stomatal status, leaf water state, cellular carbon metabolism, and etc., in drought responses [3,10]. However, little studies have been reported on the molecular bases for the stress responses in *Caragana* species at this time.

Different species of *Caragana* exhibit significantly varied abilities in drought resistance. For example, *C. rosea*, *C. microphylla*, *C. korshinskii,* and *C. kozlowii* have been evaluated for their strength of drought resistance based on leaf microstructure analysis, the drought resistance order from large to small is *C. korshinskii* > *C. microphylla* > *C. rosea* > *C. kozlowii* [11]. Furthermore, *C. microphylla* and *C. korshinskii* are closed related phylogenatically but difficult to differentiate morphologically [1,3]. Therefore, it is important to develop molecular markers to distinguish species accurately and to promote the rational use of species.

The plastome is an ideal choice for the development of molecular markers. It has many biological characteristics when compared with the nuclear genome, such as uni-parental inheritance, simpler structure, and being easier to obtain. Moreover, it provides more genetic information than a single gene/locus, resulting in much higher resolution in distinguishing closely related species. Furthermore, the plastome contains a series of genes that are related to photosynthesis, and the photosystem II (PSII) is a key part of drought stress, high temperature, and many other stresses [12,13]. Leaf physiological characteristics, such as photosynthetic capacity, which is related to the plastome function, and stomatal conductance, are the fixed indicators of water-use efficiency [14]. The water-use efficiency is critical for plants to cope with drought stress [15]. Therefore, a comparative analysis of the plastome of *Caragana* would shed light into the molecular bases for their tolerance drought.

Here, the complete plastome of *C. rosea* was sequenced and analyzed, which complements the plastid genome database of environmental stresses-resistant plants. Comparative analyses of the plastomes from *C. rosea* and other three *Caragana* species e.g., *C. korshinskii*, *C. microphylla,* and *C. kozlowii* were performed. The results that were obtained here provided valuable resources to illustrate molecular mechanisms that are related to drought resistance of *C. rosea*, to carry out chloroplast genetic engineering experiments and to select for plant individuals with favorable characteristics using molecular breeding. Furthermore, the results also provide useful information for future phylogenetic and taxonomic studies in the *Caragana* species.

## 2. Results

### 2.1. General Features of the Plastome

**Figure 1:**
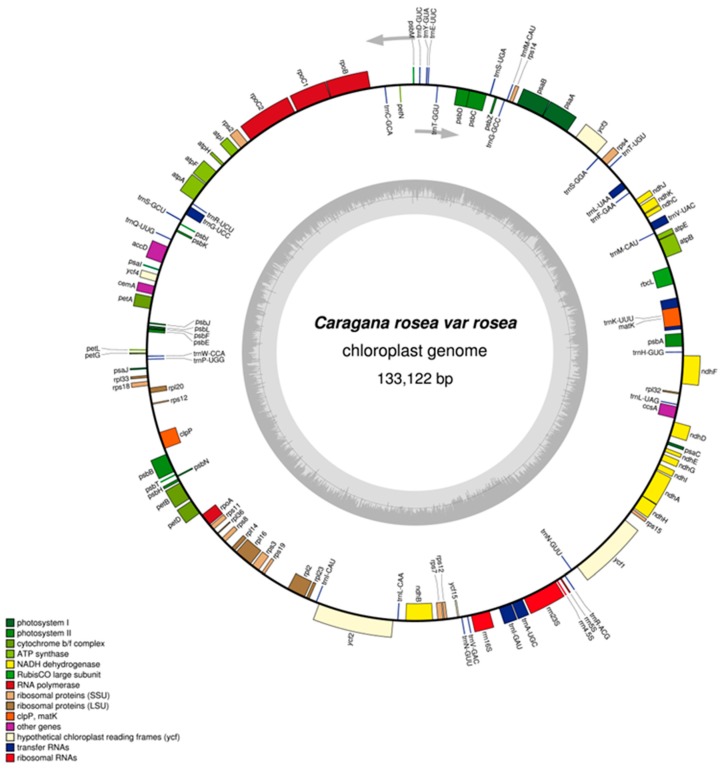
Circular gene map of the *C. rosea* plastome. Genes drawn inside the circle are transcribed clockwise, and those outside the circle are transcribed counterclockwise. Genes belonging to different functional groups are color codes. The inner circle shows the GC content. The two grey arrows represent the direction of transcription.

The complete plastome of *C. rosea* var. *rosea* is 133,122 bases in length and it lacks inverted repeat (Figure 1). This genome has been deposited in GenBank (accession number: MF593790). In the legume family, the phenomenon of IR regions loss was commonly found [16,17]. The sequence of protein-coding, tRNA, and rRNA regions accounted for 49.76%, 1.77%, and 3.4% of the whole genome, respectively; and, the rest are intergenic regions (Table 1). Moreover, a total of 111 unique genes were annotated, including 77 protein-coding, 30 tRNA, and four rRNA genes. The functional classification of these genes is shown in Table S1. The *C. rosea* plastome has 16 intron-containing genes, including 10 protein-coding genes and six tRNA genes. A total of 10 genes have only one intron, and only the *ycf3* contains two introns (Table S2). The *trnK-UUU* has the largest intron (2485 bases), which contains the *matK* gene. The *rps12* gene, which has the intron in the plastomes of other legume species [18], does not have the intron in the plastome of *Caragana*. Previous studies have shown that the loss of *rps12* intron may have occurred after the loss of the IR [19].

The overall GC content of the *C. rosea* plastome is 34.84%, whereas that for the protein-coding regions is 37.13%. The GC contents for the first, second, and third codon position with the protein-coding regions are 45.36%, 37.62%, and 28.41%, respectively. A bias towards a higher AT representation at the third codon position has also been observed in other land plant plastomes [20,21,22]. The 77 protein-coding genes comprise 66,243 bases coding for 22,081 codons. Among these codons, 2336 (10.58%) encode leucine, whereas just 258 (1.17%) encode cysteine, which are the most and least frequently amino acid in *C. rosea* plastome, respectively. The 30 unique tRNA genes include all the 20 amino acids required for protein biosynthesis (Table S3). However, there are 61 codons (excluding the three stop codons) that are found in the coding sequence (CDs) of the plastome, Since 31 of them do not have the corresponding tRNAs, the translation of their amino acids have to depend on tRNAs encoded in the nuclear genome.

The basic characteristics of cp genome from *C. rosea* and other three *Caragana* species (*C. microphylla*, *C. kozlowii*, and *C. korshinskii*) are shown in Table 1. As shown, the lengths of the four genomes are quite different, ranging from 129,331 bp to 133,122 bp. However, the length of the coding sequence differs by only 12 bp; and, the sizes of the tRNA genes and rRNA genes are also very similar. This suggests that the difference in the length of the cp genomes is caused by those of the intergenic spacers (IGS). In addition, the gene numbers, gene types, and GC contents in the four cp genomes are very similar.

### 2.2. Gene Loss Analysis

Chloroplast gene losses were analyzed between IRLC (inverted-repeat-lacking clade) of Papilionoideae in 34 species in detail (Table 2). The species are arranged in the same order as they are shown in the phylogenetic tree (see below). The *rpl22* gene and the *rps16* gene were both absent in all plastome. Moreover, the gene *rpl22* and *rps16* have been lost in most members of the angiosperm [23,24]. The two genes, which are essential for plant survival, have been transferred to the nucleus to maintain the plant’s photosynthetic capacity [25,26]. In addition, only two (*Wisteria floribunda* and *Wisteria sinensis*) plastomes possess the gene *ycf15*. The *ycf15* gene belongs to the PFAM protein family PF10705 and its function is unknown. In fact, in some plant species, the *ycf15* gene may not produce any protein because of a premature stop codons in the coding sequences of these species [27]. Because the *ycf15* genes in the GenBank are highly variable in terms of gene length and sequence, it has been difficult to annotate this gene. The absence of the *ycf4* gene occurs in many species of Papilionatae [23,28,29], whereas the plastome of four *Caragana* species contains this gene. *Psal*, *ycf1*, *rpl23*, *rps18,* and *ndhB* genes were absent in 5, 4, 3, 3, 2 species, respectively, and the *rps32* gene was also found to be transferred to nuclear genomes from plastomes in several species [30,31]. The losses of *accD*, *atpE*, *ndhA*, *psbJ*, *psbL*, *psbZ,* and *rps2* were only found in *Trifolium boissieri*, *Astragalus mongholicus* var. *nakaianus*, *Medicago falcate*, *Lathyrus littoralis*, *Medicago falcate*, *Lens culinaris,* and *Medicago falcate*, respectively; the deletion of these genes rarely occurs in the plastome of angiosperms [23]. Overall, the losses of genes are monophyletic. However, exceptions can be found in the *Medicago falcate* and several species in the genus *Lathyrus*, including *L. sativus*, *L. odoratus*, *L. inconspicuus*, *L. tingitanus, L. davidii,* and *L. pubescens.*

### 2.3. Repeat Analysis

Repeated units play an important role in genome evolution, such as structural rearrangements and size evolution [32,33]. We analyzed the content and distribution of repeated sequences in the *C. rosea* plastomes. A total of 19 repeated elements that were longer than 30 bases were identified. The similarities between all the repeated elements were greater than 90%. Tandem, forward, and palindromic repeats presented a similar pattern of distribution. Most of them were found in the intergenic spacer region (IGS), around 31.6% were found in the protein-coding region, and only one repeat was found in the tRNA gene. However, the length of repeats among *C. rosea* plastome was obviously longer than those in other legumes [24,34], the largest repeat unit was 291 base long and was located in the spacer between the genes *rps12* and *clpP.* The type, location, and sequence of the repeat units are shown in Table S4.

More remarkably, we found that 31.6% and 21.1% of these repeats were located in the IGS(*rps12-clpP*) and IGS(*rps19-rpl2*) regions, with the total length of the IGS regions being 2139 bases and 4060 bases, respectively. By contrast, the other three *Caragana* species do not have so many repeat units in the same regions. For example, the length of the corresponding IGS(*rps12-clpP*) regions in *C. microphylla* and *C. korshinskii* are less than 1 kb. Repeats are known to play a major role in plastome size evolution in angiosperm [35], the presence of abundant repeat sequences may be the reason why the genome of *C. rosea* is larger than the other three *Caragana* species.

**Figure 2:**
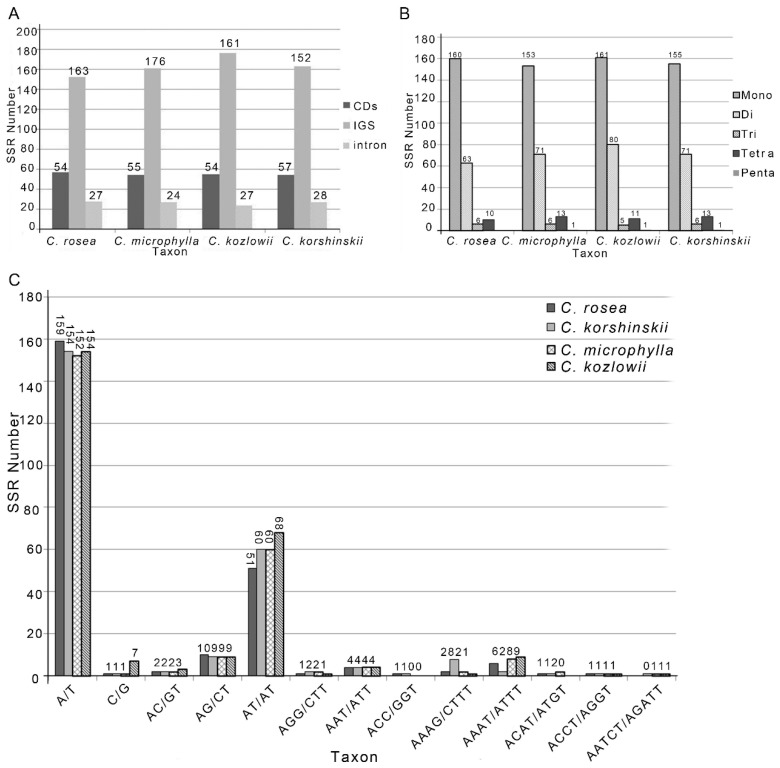
Statistics of simple sequence repeat (SSRs) detected in the plastome of four *Caragana* species. (**A**) Numbers of SSRs found in the coding (CDS), intergenic (IGS), and intronic regions, respectively; (**B**) number of different SSR types identified in the four genomes; and, (**C**) number of identified SSR motifs in different repeat class types.

Simple sequence repeat (SSR) loci are effective molecular markers because of their high variability that are wide distribution throughout the whole plastome [36]. SSR can provide useful information in polymorphism investigations and population genetics [37,38]. We identified SSRs in the plastome of four *Caragana* species. The number of SSRs ranges from 239 to 258 (Figure 2). In *Caragana rosea*, 66.9% are mono-nucleotide repeats, as compared with only 26.4%, 2.5%, and 4.2%, of di-, tri-, and tetra-nucleotide repeats, respectively. Of these SSR loci, 159 contained A or T, whereas only one had G or C; similarly, most dinucleotide repeat sequences were composed of AT/AT repeats. This result is consistent with previous reports that most of the SSR in plastomes are composed of short polyA or polyT repeats, while tandem repeats of G or C are rare [39]. We also analyzed the occurrence of SSRs in the CDs and found that there are fewer SSRs in the protein-coding regions than in the non-coding regions. The plastomes of the other three *Caragana* species are similar to that of *C. rosea* in terms of the number, distribution, and the GC contents of the SSRs.

### 2.4. Sequence Divergence Analysis among Caragana Species

**Figure 3:**
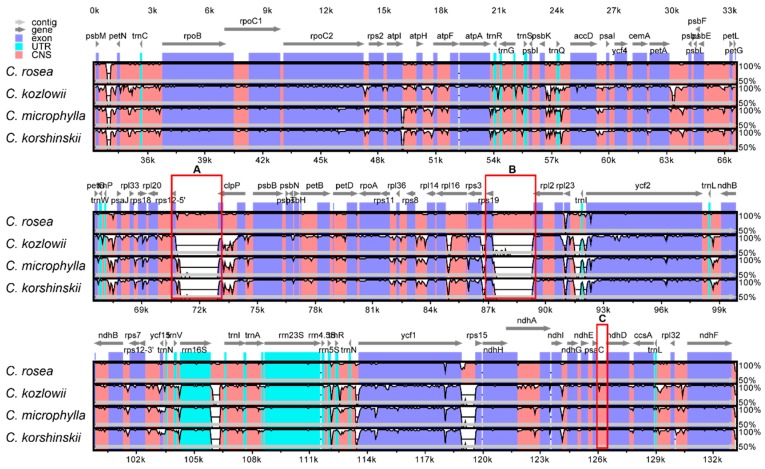
Structure comparison of the four plastomes by using the mVISTA program. Gray arrows and thick black lines above the alignment indicate genes with their orientation and the position of the IRs, respectively. A cut-off value of 70% identity was used for the plots, and the Y-scale represents the percent identity between 50% and 100%. UTR: Untranlated Regions; CNS: Conserved Non-coding Sequences. A: IGS(*rps12*-*clpP*); B: IGS(*rps19*-*rp12*); C: IGS(*psaC*-*ndhD*).

To elucidate the level of sequence similarities between the *C. rosea* and the other three *Caragana*, the plastome sequences were compared while using the annotated *C. rosea* plastome as the reference (Figure 3). As shown, the four plastome sequences are highly similar. However, there are significant differences between *C. rosea* and other three *Caragana* species in some IGS, such as IGS(*rps12*-*clpP*) (square A), IGS(*rps19*-*rpl2*) (square B), and etc., which may be related to the unique large repeat fragments in the *C. rosea* plastome. For the regions IGS(*psaC*-*ndhD*) (square C), the sequence of *C. rosea* plastome is highly similar to those of *C. microphylla* and *C. korshinskii*, but it is quite different from that of *C. kozlowii*. Overall, the protein-coding regions are highly conserved, while the non-coding regions have different degrees of divergence between the *C. rosea* plastome and those of the other three. This suggests that the IGS of *Caragana* species has evolved rapidly.

**Figure 4:**
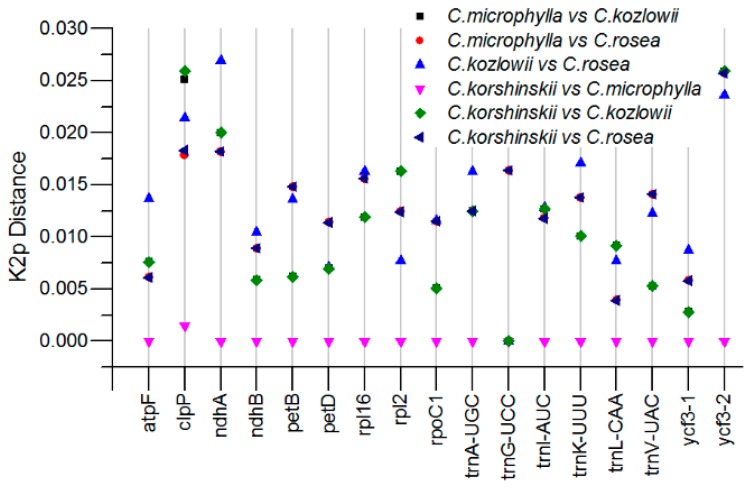
K2p distances for introns among *C. rosea*, *C. microphylla*, *C. kozlowii*, and *C. korshinskii*.

Highly variable sites in the genome can be used to develop molecular markers. We set out to identify the highly variable sites. We conducted pairwise distance comparison analysis for each non-coding region, including the intergenic region and the intron region using Kimura 2-parameter (K2p) model to identify divergence hotspot regions among the *Caragana* species. As expected, the variation of the intron sequence is relatively low, and the K2p distances ranged from 0.000 to 0.0269 (Figure 4, Table S5). The *clpP*, *ndhA* introns and the second intron of *ycf3* show the highest K2p values among the four *Caragana* species.

**Figure 5:**
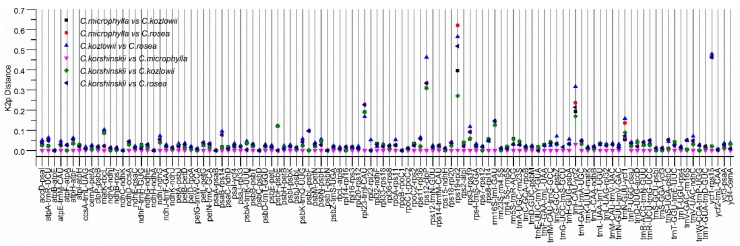
K2p distances for IGS regions among *C. rosea*, *C. microphylla*, *C. kozlowii*, and *C. korshinskii*.

For IGSs, the K2p values ranged from 0 to 0.6207 (Figure 5). As described in the method section, we calculatecd the mean+2*STD (Standard Deviation) as the threshold for a IGS to be highly variable. It is 0.1649 in this case. The sequence divergences of the IGS regions ranged from 0 to 0.395 between *C. microphylla* and *C. kozlowii*, ranged from 0 to 0.6207 between *C. microphylla* and *C. rosea*, ranged from 0 to 0.5641 between *C. kozlowii* and *C. rosea*, ranged from 0 to 0.068 between *C. korshinskii* and *C. microphylla*, ranged from 0 to 0.3094 between *C. korshinskii* vs C. kozlowii, and ranged from 0 to 0.5176 between *C. korshinskii* vs *C. rosea.* The seven IGS regions (*atpB*-*atpE*, *ndhC*-*ndhK*, *ndhH*-*ndhA*, *psaA*-*psaB*, *psbC*-*psbD*, *psbT*-*psbN*, and *rpoB*-*rpoC1*) are 100% identical with the K2p values of 0. Meanwhile, we found that the K2p values were particularly high for the five IGS regions: *rpl23*-*trnI*-*CAU*, *rps12*-*clpP*, *rps19*-*rpl2*, *trnI*-*CAU*-*ycf2*, and *ycf1*-*rps15*.

The high degree of similarity among the plastomes of *C. korshinskii* and *C. microphylla* is consistent with their high degree of similarity in morphological characteristics. To determine whether these species can be distinguished with common molecular markers, such as ITS, *rbcL,* and *matK*, we compared the sequences of these markers from these species. It is found that these regions could not be used to distinguish the two confusable species with 99%, 100%, and 100% identities among these marker sequences, respectively. Interestingly, two regions IGS(*trnI*-*CAU*-*ycf2*) and IGS(*trnN*-*GUU*-*ycf1*) from *C. korshinskii* and *C. microphylla* have large K2p distances, indicating a high degree of sequence divergences. Moreover, these two regions have relatively high K2p values in the pairwise distance comparison analysis of these four species, which can be used to develop novel molecular markers to distinguish the four *Caragana* species accurately.

### 2.5. Phylogenetic Analysis

**Figure 6:**
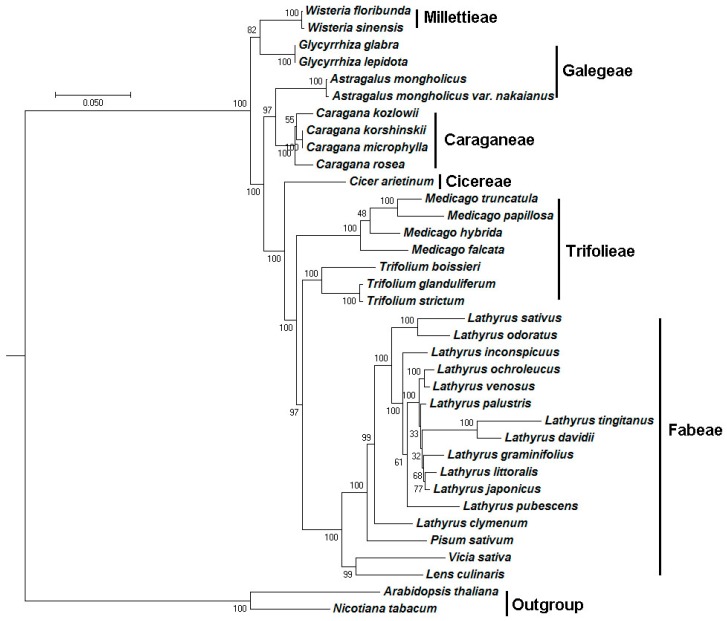
Molecular phylogenetic analyses of plastomes in the inverted-repeat-lacking clade of Papilionoideae. The tree was constructed with the sequences of 63 proteins present in all 36 species by using the maximum likelihood method implemented in RAxML. Bootstrap supports were calculated from 1000 replicates. *Nicotiana tabacum* and *Arabidopsis thaliana* were set as outgroups.

The plastome sequence is an important resource for studying phylogenetic relationships and taxonomic status in the angiosperm [28]. In order to determine the phylogenetic position of *Caragana* in the Papilionoideae, we conducted multiple sequence alignments while using 63 common protein sequences from the plastomes of 36 species. *Arabidopsis thaliana* and *Nicotiana tabacum* were set as outgroup. The other 34 species contained six plant families that belong to the IRLC of Papilionoideae, including *Galegeae* (4), *Caraganeae* (4), *Cicereae* (1), *Fabeae* (16), *Trifolieae* (7), and *Millettieae* (2). The numbers in the parentheses represent the number of species in the corresponding taxa. The final dataset comprised 18745 positions and were subjected to phylogentic analysis using RaxML. Without surprise, *C. rosea* is found to locate in the same branch as the other three *Caragana* species, with 100% bootstrap values (Figure 6).

### 2.6. Selective Pressure Analysis

As synonymous substitutions accumulate nearly neutrally, non-synonyous substitutions are subject to selective pressures of varying degree and direction (positive or negative). In general, the ratio of nonsynonymous to synonymous substitution (ω) measures the levels of selective pressure operating in a protein coding gene. To test which genes were subject to positive selection at the *Caragana* branch, we conducted the selection analysis of the exons of each protein-encoding gene using the adaptive Branch-Site Random Effects Likelihood (aBSREL) model. A total of 69 branches among 36 species (listed in Section 2.5) were tested for diversifying selection. Significance was assessed using the Likelihood Ratio Test at a threshold of *p* ≤ 0.05, after correcting for multiple testing.

Five genes (*rpl16*, *rpl20*, *rps11*, *rps7,* and *ycf1*) were found to have evolved under positive selection in *Caragana* branch in the phylogeny, the significance and number of rate categories inferred at the *Caragana* branch are provided in Table 3. The optimized branch length of the *Caragana* branch is 0.0128. The genes can be classified into two groups. The first group contains four ribosomal protein-coding genes. There is very strong evidence in selective pressure for the *rpl20* and *rps11* genes: positive selection was detected in 16 branches for both genes. In contrast, four branches (*Medicago hybrida*, *Medicago papillosa*, *Medicago falcata,* and *Caragana*) have experienced positive selection for the *rpl16* gene. Two branches (*Lathyrus clymenum*, *Caragana*) excluding those for the outgroup have experienced positive selection for the *rps7* gene, with two rate classes per branch. The second group containd the *ycf1* gene. The analysis did not include four species (*Lens culinaris*, *Wisteria floribunda*, *Wisteria sinensis*, and *Medicago falcata*) lacking the *ycf1* gene. The aBSREL model selection procedure identifies 42 branches for *ycf1* to have been significantly selected.

To find out which sites were subject to positive selection along the *Caragana* branch, codeml from PAML (v4.9) were used to analyze the Ka/Ks for the four genes: *rpl16*, *rpl20*, *rps11,* and *rps7*, using the branch-site model. The *Caragana* branch shown in Figure 6 were set as the foreground branch and all the other branches were set as the background branches. It is found that two sites: 36Y and 67Q was potentially under positive selection for the *rpl16* gene along the *Caragana* branch. In contrast, one site: 97Y were potentially under positive selection for the *rpl20* gene. Seven sites: 32K, 72T, 86N, 92V, 97Q, 103I, 137T were potential positively selected for the *rps11* gene. Four sites: 59E, 60T, 65V, 132V were positively selected for the *rps7* gene. As no three-dimensional (3D) structures are available for *Caragana* proteins, we cannot determine the functional and evolutionary significance of these sites at this time.

## 3. Discussion

*Caragana* species are xeromorphic, heat-resistant, and cold-resistant plants. Understanding the underlying molecular mechanism of this genus is of great interest for molecular breeding. As sessile organisms, plants must cope with abiotic stresses, such as soil salinity, drought, and extreme temperatures. Stress signaling pathways in plants have been extensively studied and reviewed [7,40]. All of these signaling pathways involve protein kinases that are related to yeast SNF1 and mammalian AMPK, suggesting that stress signaling in plants evolved from energy sensing.

Signals that are caused by limited water (drought stress) or excessive salt (salt stress) can be divided into primary and secondary types. The primary signal caused by drought is also called hyperosmotic stress. Salt stress exerts both osmotic and ionic effects on cells. The secondary effects are rather complex and they include oxidative stress; such effects include damage to cellular components (such as membrane lipids, proteins, and nucleic acids), chloroplast, mitochondria, ER, and metabolic dysfunction [7].

Chloroplast is an organelle where photosynthetic electron transport and many metabolic reactions occur. Environmental stress can easily perturb the metabolic balance in chloroplasts. The disturbance in chloroplast homeostasis is then passed to the nucleus through retrograde signals; as such, all cellular activities can be adjusted and coordinated. The chloroplast is a major site for the production of reactive oxygen species (ROS), such as superoxide anion, hydrogen peroxide, hydroxyl radical, and singlet oxygen [41]. Various environmental stresses, particularly high light stress, can exacerbate ROS production, thereby disrupting ROS-managing systems and generating various secondary messengers.

The relationship between protein synthesis and stress response has been studied [7]. The accuracy of protein synthesis is critical for life because a high degree of fidelity of the translation of the genetic information is required to accomplish the needs for cellular functions and to preserve variability developed by evolution. Even in simple organisms, this process involves more than 100 macromolecules, such as ribosomal proteins, translation factors, aminoacyl-tRNA synthetases, ribosomal RNAs, and transfer RNAs. Moreover, in vivo or in vitro experiments indicated that several macromolecules that participate in translation are the targets of oxidation; hence, translation is directly targeted by oxidative species. In bacteria, target macromolecules include elongation factors, such as Tu [42,43], Ts (EF-Ts) [44], and G (EF-G) [45,46,47,48]; several ribosomal proteins [49,50,51,31,36,37]; tRNAs [38,39,40,41,42,52,53]; and, aminoacyl-tRNA synthetases (aaRS) [43,49,50,51,54]. In particular, the disulfide bond formation of ribosomal proteins S7 and L16 are affected by oxidative stress [51]. Furthermore, scholars have identified the covalent binding of ribosomal protein S11 to chaperon/oxido-reductase protein through cysteine bond [43].

Previous studies showed that environmental stress can cause oxidative stress, which in turn affects the translation system, particularly in prokaryote originated systems, such as chloroplast. When considering the axis of environmental stress->oxidative stress->translation system, we can speculate that ribosomal protein-coding genes, such as *rps7*, *rps11*, *rpl16*, and *rpl20*, are strongly selected to maintain the integrity of the protein synthesis machinery under various environmental stresses. This phenomenon might, at least in part, contribute to the strong environmental stress resistance characteristics of the Caragana species. Additional analyses would be needed to confirm this hypothesis.

## 4. Materials and Methods

### 4.1. Plant Material, DNA Extraction, and Sequencing

The fresh leaves of the *C. rosea* were collected from the Institute of Medicinal Plant Development, China. After washing, the leaves were kept in the −80 refrigerator until use. DNA from about 100 mg leaves was extracted using the modified CTAB (Cetyltrimethylammonium bromide) method. Subsequently, the extracted DNA integrity and concentration were detected by electrophoresis in 1% (*w/v*) agarose gel and spectrophotometer (Nanodrop 2000, Thermo Fisher Scientific, Waltham, MA, USA). The genomic DNA of *C. rosea* was subjected to high-throughput sequencing using an Illumina Hiseq2000 sequencer (Illumina Inc., San Diego, CA, USA), with insert sizes of 500 bases for the library.

A total of 18,932,846 paired-end reads were obtained with 100 bases long. The other three plastome sequences of *Caragana* e.g., *C. korshinskii* (Accession number: NC_035229), *C. kozlowii* (Accession number NC_035228), *C. microphylla* (Accession number NC_032691), and ITS (Accession number: FJ537266, FJ537264) were obtained from Genbank.

### 4.2. Genome Assembly and Gap Filling

In order to extract the reads belonging to the plastome from those for the total DNAs, we downloaded 1688 plastome sequences from GenBank in February 2016, which were used to search against Illumina paired-end reads using BLASTN with an E-value cutoff of 1 × 10^5^ [55]. The genome sequence of *C. kozlowii* was found to have the highest similarity and it was chosen as the reference sequence for the following assembly.

A total of 3076 paired-end reads similar to the *C. kozlowii* plastome sequence were selected and assembled by AbySS (v1.5.2) [56] and CLC Genomics Workbench (v7) Software. Nine and seven contigs were obtained using the two software tools, respectively. Then, the 16 sequences were further assembled by the Seqman module of DNAStar (v6.10.01), resulting in three contigs. The gaps between the contigs were filled with PCR amplification and Sanger sequencing using the sequence-specific primers (Table S6) designed to cross the gaps. Finally, the draft plastome sequence was validated by mapping the raw Illumina paired-end reads against it using Bowtie2 (v2.0.1) with default settings [57].

### 4.3. Genome Annotation and Characteristics Analysis

The *C. rosea* plastome sequence was annotated by CpGAVAS web service [58], with the default parameter. The tRNA genes were annotated using ARAGORN [59] and tRNAscan-SE [60]; the protein sequences were verified again by BLASP against the GenBank sequences. Subsequently, the intron/exon boundaries and the start/stop codons of predicted genes are manually edited using the Apollo program (v1.11.8) [61]. The circular plastome map of *C. rosea* was drawn using OrganellarGenomeDRAW [62]. Both GC contents and codon usage were calculated using the programs Cusp and Compseq from EMBOSS (v6.3.1) [63].

### 4.4. Repeat and SSR Analysis

Repeats (palindrome and forward repeats) were identified by REPuter web service [64], with the settings of 3 for the Hamming Distance (sequence identity ≥ 90%) and 30 for Minimal Repeat Size, as reported previously [34,65]. The number and location of tandem repeated elements in the *Caragana* genus plastome were determined using the Tandem Repeats Finder [66], with the following parameters: matches, mismatches and indels, minimum alignment score, and the maximum period size were 2, 7, 50, and 500, respectively. We manually verified all of the detected repeats and removed nested and redundant sequences. SSR in the plastome was analyzed by MISA software with the same parameters as reported previously [67]. Briefly, the cutoff for the numbers of units for mono-, di-, tri-, tetra-, penta-, and hexa-nucleotides were 8, 4, 4, 3, 3, and 3, respectively.

### 4.5. Comparative Genomic Analysis

The complete plastome sequence of *C. rosea* was compared with the those of *C. korshinskii*, *C. kozlowii*, and *C. microphylla*, using the mVISTA program in a Shuffle-LAGAN mode with default parameters [68]. The annotated *C. rosea* plastome was used as the reference. In order to analyze sequence diversity and selective pressure, a total of 112 intergenic regions, 17 introns, and 76 exons were extracted from the four plastomes using custom MatLab scripts. The corresponding nucleotide sequences were aligned using the CLUSTALW2 (v2.0.12) program with options “-type = DNA -gapopen = 10 -gapext = 2” [69]. Pairwise distance were determined with the Distmat program that was implemented in EMBOSS (v6.3.1) [63] using the Kimura 2-parameters (K2p) evolution model [70] for intergenic regions and introns. To determine the threshold for the K2p distance to be highly variable, we calculated the mean and the standard deviation for all the K2p values. The mean + 2*STD were then set as the threshold. The 76 exons sequences were aligned using the RevTrans (v2.0) [71] with the option of CLUSTALW2 program. Subsequently, the selective pressure analysis were conducted using adaptive branch-site random effects likelihood (aBSREL) model [72], implemented in HyPhy (https://veg.github.io/hyphy-site/getting-started/#characterizing-selective-pressures). Finally, we analyzed which sites were subject to positive selection along *Caragana* branch using the Codeml program that was implemented in PAML (v4.9) [73].

### 4.6. Phylogenetic Analysis

The plastome sequences of 33 species belonging to the IRLC (inverted-repeat-lackingclade) of Papilionoideae and two outgroup species (*Arabidopsis thaliana* and *Nicotiana tabacum*) were downloaded from NCBI RefSeq database, and a total of 63 protein sequences that were present in all of the 35 species and *C. rosea* were obtained by manual detection (ATPA, ATPB, ATPF, ATPH, ATPI, CCSA, CEMA, CLPP, MATK, NDHC, NDHD, NDHE, NDHF, NDHG, NDHH, NDHI, NDHJ, NDHK, PETA, PETB, PETD, PETG, PETL, PETN, PSAA, PSAB, PSAC, PSAJ, PSBA, PSBB, PSBC, PSBD, PSBE, PSBF, PSBH, PSBI, PSBK, PSBM, PSBN, PSBT, RBCL, RPL14, RPL16, RPL2, RPL20, RPL32, RPL33, RPL36, RPOA, RPOB, RPOC1, RPOC2, RPS11, RPS12, RPS14, RPS15, RPS19, RPS3, RPS4, RPS7, RPS8, YCF2, and YCF3) (Table S7). For the phylogenetic analysis, these protein sequences were aligned using the CLUSTALW2 (v2.0.12) program with options “-gapopen = 10 -gapext = 2 -output = phylip”. The Maximum Likelihood method implemented in RaxML (v8.2.4) [69] was used to inferred the evolutionary history, using “raxmlHPC-PTHREADS-SSE3 -f a -N 1000 -m PROTGAMMACPREV -x 551314260 -p 551314260 -o A_thaliana, N_tabacum -T 20”. Subsequently, the Bootstrap analysis was also performed with 1000 replicates for the phylogenetic tree.

## 5. Conclusions

In this study, we sequenced the plastome of *C. rosea* and carried out a comparative study with those from *C. microphylla*, *C. kozlowii*, and *C. korshinskii*. Phylogenetic analyses showed that four *Caragana* species were on a monophyletic clade, with 100% bootstrap values. Analyses of selective pressure revealed that five genes: *rpl16, rpl20*, *rps11, rps7,* and *ycf1* were evolved undergoing positive selection. Analyses of sequence divergence found two sites: IGS(*trnI-CAU-ycf2*) and IGS(*trnN-GUU-ycf1*) had high degree of variations and might be sources for markers that can be used to distinguish these four species. The results presented in this paper will facilitate the further investigation for these four species in terms the molecular mechanisms for drought-resistance.

## Figures and Tables

**Table 1:** Characteristics of *Caragana* plastome.

Plastome Characteristics	*C. rosea*	*C. microphylla*	*C. kozlowii*	*C. korshinskii*
complete genome length	133,122 bp	130,029 bp	131,274 bp	129,331 bp
No.of unique genes	110	110	110	110
No.of unique protein-coding genes	76	76	76	76
No.of unique tRNA genes	30	30	30	30
No.of unique rRNA genes	4	4	4	4
Size of protein-coding genes	66,243 bp (49.76%)	66,231 bp (50.94%)	66,234 bp (50.45%)	66,231 bp (51.21%)
Size of tRNA genes	2359 bp (1.77%)	2370 bp (1.82%)	2285 bp (1.74%)	2370 bp (1.83)
Size of rRNA genes	4537 bp (3.4%)	4520 bp (3.48%)	4521 bp (3.44%)	4520 bp (3.49)
Overall GC contents	34.84%	34.26%	34.50%	34.36%
GC contents of protein-coding genes	Coding GC 37.13%	Coding GC 36.88%	Coding GC 37.03%	Coding GC 36.88%
	#1st position 45.36%	#1st position 44.98%	#1st position 45.30%	#1st position 44.96%
	#2nd position 37.62%	#2nd position 37.67%	#2nd position 37.58%	#2nd position 37.67%
	#3rd position 28.41%	#3rd position 27.99%	#3rd position 28.21%	#3rd position 27.99%
GC contents of tRNA genes	52.73%	53.14%	53.15%	53.05%
GC contents of rRNA genes	54.77%	54.82%	54.75%	54.82%

**Table 2:** Gene losses in the plastomes from the inverted-repeat-lacking clade (IRLC) of Papilionoideae.

Category	Name of Species	*rps16*	*rpl22*	*ycf15*	*ycf4*	*psaI*	*ycf1*	*rpl23*	*rps18*	*ndhB*
*Millettieae*	*W. floribunda*	−	−	+	+	+	−	+	+	+
	*W. sinensis*	−	−	+	+	+	−	+	+	−
*Galegeae*	*G. glabra*	−	−	−	+	+	+	+	+	+
	*G. lepidota*	−	−	−	+	+	+	+	+	+
	*A. mongholicus*	−	−	−	+	+	+	+	+	+
	*A. mongholicus var. nakaianus*	−	−	−	+	+	+	+	+	+
*Caraganeae*	*C. kozlowii*	−	−	−	+	+	+	+	+	+
	*C. korshinskii*	−	−	−	+	+	+	+	+	+
	*C. microphylla*	−	−	−	+	+	+	+	+	+
	*C. rosea var. rosea*	−	−	−	+	+	+	+	+	+
*Cicereae*	*C. arietinum*	−	−	−	−	+	+	+	+	+
*Trifolieae*	*M. truncatula*	−	−	−	−	+	+	+	+	+
	*M. papillosa*	−	−	−	−	+	+	+	+	+
	*M. hybrida*	−	−	−	−	+	+	+	+	+
	*M. falcata*	−	−	−	+	+	−	+	+	−
	*T. boissieri*	−	−	−	−	+	+	+	+	+
	*T. glanduliferum*	−	−	−	−	+	+	+	+	+
*Fabeae*	*T. strictum*	−	−	−	−	+	+	+	+	+
	*L. sativus*	−	−	−	+	−	+	−	+	+
	*L. odoratus*	−	−	−	−	−	+	+	−	+
	*L. inconspicuus*	−	−	−	−	−	+	+	+	+
	*L. ochroleucus*	−	−	−	−	+	+	+	+	+
	*L. venosus*	−	−	−	−	+	+	+	+	+
	*L. palustris*	−	−	−	−	+	+	+	+	+
	*L. tingitanus*	−	−	−	−	+	+	+	−	+
	*L. davidii*	−	−	−	−	−	+	+	+	+
	*L. graminifolius*	−	−	−	−	+	+	+	+	+
	*L. littoralis*	−	−	−	−	+	+	+	+	+
	*L. japonicus*	−	−	−	−	+	+	+	+	+
	*L. pubescens*	−	−	−	−	−	+	+	+	+
	*L. clymenum*	−	−	−	−	+	+	+	+	+
	*P. sativum*	−	−	−	−	+	+	−	+	+
	*V. sativa*	−	−	−	−	+	+	−	+	+
	*L. culinaris*	−	−	−	−	+	−	+	−	+
	Number ^a^	34	34	32	22	5	4	3	3	2

^a^ The number refers to the total number of species that do not have the gene. “+”: presence; “−” absence.

**Table 3:** The results of positive selection genes at *Caragana* branch.

Gene	B	LRT	Test *p*-Value	Uncorrected *p*-Value	ω Distribution over Sites
*rpl16*	0.0128	17.4234	0.0037	0.0001	ω1 = 0.121 (98%)
					ω2 = 781 (1.9%)
*rpl20*	0.0128	31.0401	0	0	ω1 = 0.147 (99%)
					ω2 = 290 (0.92%)
*rps11*	0.0128	12.0303	0.0452	0.0008	ω1 = 0.226 (100%)
					ω2 = 0.824 (0.20%)
					ω3 = 10000 (0.14%)
*rps7*	0.0128	24.3289	0.0001	0	ω1 = 0.00 (100%)
					ω2 = 286 (0.43%)
*ycf1*	0.0128	34.4372	0	0	ω1 = 0.384 (99%)
					ω2 = 91.3 (0.74%)

B: Optimized branch length; LRT: Likelihood ratio test statistic for selection; Test *p*-value: *p*-value corrected for multiple testing; Uncorrected *p*-value: Raw *p*-value without correction for multiple testing.

## Supplementary Materials

Supplementary materials can be found at http://www.mdpi.com/1422-0067/19/5/1419/s1.
