# Supplementary material for: Sequencing, Characterization, and Comparative Analyses of the Plastome of Caragana rosea var. rosea
Source: Int J Mol Sci. 2018 May 9;19(5):1419. doi: 10.3390/ijms19051419 (PMC5983699; doi:10.3390/ijms19051419)
Supplement: Supplementary file 1 [file ijms-19-01419-s001.pdf]

Table S1 Genes contained in the *Caragana rosea* choloplast genome.

**Category for genes**	**Group of genes**	**Name of genes**
Self replication	rRNA genes	*rrn23S,rrn16S,rrn5S,rrn4.5S*
	tRNA genes	30 trn genes(6 contain an intron)
	Small subunit of ribosome	*rps2,rps18,rps8,rps4,rps7,rps11,rps12,rps15,rps19,rps3,rps14*
	Large subunit of ribosome	*rpl14,rpl36,rpl23,rpl20,rpl32,rpl2*,rpl33,rpl16**
	DNA dependent RNA polymerase	*rpoC1*,rpoC2,rpoB,rpoA*
Genes for photosynthesis	Subunits of NADH-dehydrogenase	*ndhK,ndhJ,ndhF,ndhG,ndhE,ndhD,ndhB*,ndhC,ndhA*,ndhH, ndhI*
	Subunits of photosystem Ⅰ	*psaI,psaC,psaB,psaA,psaJ*
	Subunits of photosystem Ⅱ	*psbZ,psbJ,psbB,psbA,psbC,psbF,psbI,psbK,psbT,psbD,psbN,psbL,psbM,psbE,psbH*
	Subunits of cytochrome b/f complex	*petN,petA,petD*,petG,petB*,petL*
	Subunits of ATP synthase	*atpI,atpE,atpA,atpB,atpH,atpF**
	Large subunit of rubisco	*rbcL*
Other genes	Maturase	*matK*
	Protease	*clpP**
	Envelope membrane protein	*cemA*
	Subunit of Acetyl-CoA-carboxylase	*accD*
	c-type cytochrom synthesis gene	*ccsA*
Genes of unknown functions Open Reading Frames(ORF,ycf)		*ycf4,ycf1,ycf15,ycf3**,ycf2*

One asterisks after gene names reflects one intron containing genes, two asterisks reflects two introns. The *rps12* gene is a trans-splicing gene.

Table S2 The lengths of introns and exons for intron-containing genes.

Gene	Strand	Start	End	Exon I	Intron I	Exon II	Intron II	Exon III
trnK-UUU	-	1849	4399	37	2485	29		
trnV-UAC	+	10017	10670	39	578	37		
trnL-UAA	-	13991	14597	37	520	50		
ycf3	+	17604	19682	124	702	230	870	153
rpoC1	+	40045	42893	430	794	1625		
atpF	+	50902	52160	172	680	407		
trnG-UCC	-	54330	55078	23	677	49		
rps12-5′ end	-	70581	70709	129	-	-		
rps12-3′ end	-	102157	102414	-	-	258		
clpP	-	72969	74338	363	782	225		
petB	+	77274	78747	6	826	642		
petD	+	78952	80144	9	710	474		
rpl16	-	84340	85846	9	1093	405		
rpl2	-	89344	90860	394	653	470		
ndhB	-	99104	101265	723	677	762		
trnI-GAU	+	106585	107608	42	947	35		
trnA-UGC	+	107673	108556	38	811	35		
ndhA	+	121218	123494	553	1185	539		

Table S3 Codon usage and codon–anticodon recognition patterns in *Caragana* rosea chloroplast genome.

Amino acid	Codon	Number	Fraction	Frequency	tRNA
Ala	GCA	339	0.278	1.54%	*trnA-UGC*
Ala	GCC	182	0.149	0.82%	-
Ala	GCG	125	0.102	0.57%	-
Ala	GCU	575	0.471	2.60%	-
Cys	UGC	62	0.24	0.28%	*trnC-GCA*
Cys	UGU	196	0.76	0.89%	-
Asp	GAC	171	0.194	0.77%	*trnD-GUC*
Asp	GAU	709	0.806	3.21%	-
Glu	GAA	884	0.752	4.00%	*trnE-UUC*
Glu	GAG	291	0.248	1.32%	-
Phe	UUC	396	0.308	1.79%	*trnF-GAA*
Phe	UUU	891	0.692	4.04%	-
Gly	GGA	616	0.41	2.79%	*trnG-UCC*
Gly	GGC	123	0.082	0.56%	*trnG-GCC*
Gly	GGG	224	0.149	1.01%	-
Gly	GGU	539	0.359	2.44%	-
His	CAC	112	0.218	0.51%	*trnH-GUG*
His	CAU	401	0.782	1.82%	-
Ile	AUA	624	0.32	2.83%	*trnI-UAU*
Ile	AUC	387	0.198	1.75%	*trnI-GAU*
Ile	AUU	940	0.482	4.26%	-
Lys	AAA	914	0.768	4.14%	*trnK-UUU*
Lys	AAG	276	0.232	1.25%	-
Leu	CUA	303	0.13	1.37%	*trnL-UAG*
Leu	CUC	146	0.062	0.66%	-
Leu	CUG	135	0.058	0.61%	-
Leu	CUU	490	0.21	2.22%	-
Leu	UUA	769	0.329	3.48%	*trnL-UAA*
Leu	UUG	493	0.211	2.23%	*trnL-CAA*
Met	AUG	513	1	2.32%	*trn(f)M-CAU*
Asn	AAC	239	0.228	1.08%	*trnN-GUU*
Asn	AAU	810	0.772	3.67%	-
Pro	CCA	274	0.301	1.24%	*trnP-UGG*
Pro	CCC	179	0.197	0.81%	-
Pro	CCG	101	0.111	0.46%	-
Pro	CCU	356	0.391	1.61%	-
Gln	CAA	627	0.8	2.84%	*trnQ-UUG*
Gln	CAG	157	0.2	0.71%	
Arg	AGA	378	0.302	1.71%	*trnR-UCU*
Arg	AGG	129	0.103	0.58%	-
Arg	CGA	292	0.233	1.32%	-
Arg	CGC	83	0.066	0.38%	-
Arg	CGG	91	0.073	0.41%	-
Arg	CGU	279	0.223	1.26%	*trnR-ACG*
Ser	AGC	103	0.063	0.47%	*trnS-GCU*
Ser	AGU	351	0.214	1.59%	-

Ser	UCA	327	0.199	1.48%	*trnS-UGA*
Ser	UCC	240	0.146	1.09%	*trnS-GGA*
Ser	UCG	155	0.094	0.70%	-
Ser	UCU	468	0.285	2.12%	-
Thr	ACA	353	0.313	1.60%	*trnT-UGU*
Thr	ACC	201	0.178	0.91%	*trnT-GGU*
Thr	ACG	109	0.097	0.49%	-
Thr	ACU	465	0.412	2.11%	-
Val	GUA	445	0.367	2.02%	*trnV-UAC*
Val	GUC	152	0.125	0.69%	*trnV-GAC*
Val	GUG	157	0.13	0.71%	-
Val	GUU	458	0.378	2.07%	-
Trp	UGG	371	1	1.68%	*trnW-CCA*
Tyr	UAC	151	0.183	0.68%	*trnY-GUA*
Tyr	UAU	676	0.817	3.06%	-
Stop	UAA	38	0.487	0.17%	-
Stop	UAG	19	0.244	0.09%	-
Stop	UGA	21	0.269	0.10%	-

Table S4 Distribution of large repeat loci in the *Caragana rosea* choloplast genome.

**Repeat Number**	**Repeat Size(bp)**	**Type**	**position1**	**position2**	**position3**	**Location**	**Repeat Unit sequence**
1	291	T	71622	71923	-	IGS(rps12-clpP)	TATTGATCAGTGTCAGTACTATTACTGACACTGATCTTGAATTTTGTATATCAAATTCTATTCATTTACTCTC ACACTATTTACCGCCGAACCAACGCCTATACACTTCGATAACTAAAACAGAACCACAAATCACTGCGTTT AATCCTTTCAAGAAATTCTCTGTGGTGAATCCTTTCAGGACTTTTAAGATAAGTTTTTTCATATAATAGTTG ACCCCCTCCTTGGGTTTTGTTTACAAAATAAATAAAATAAGAAGAAAATATTCTTTTGAAAATGATCGAG AATGAA(X2)
2	47	T	86614	86661	86708	CDS(rps3),IGS(rps3-rps19)	ACCAAGTCTGAAACCAAGTGGATTTATTTTTTGTCCCATAATCCCTC(X3)
3	61	T	88203	88266	-	IGS(rps19-rpl2)	CCAAGTCAATTTGTTGAATGTGATA--TGTTGACCTCCATTTATGTATAGCTAAACGAACAAG(X2)
4	89	T	90879	90968	-	CDS(rpl2),IGS(rpl2-rpl23)	ATTTAAGTTCGTTTCTTTCTAAGAGGTGGAATAGAATAACCCGGTTGAAGCGTAATGATCATACGTTTGTA AAGCATTGTATGTCCCAT(X2)
5	62	T	91907	91969	-	IGS(trnI-CAU-ycf2)	TGAATAACCTAATTCCAATTGAAGTGAAATCTTTTGGATAAGTCAAGAGAAATCAATACAAT(X2)
6	33	T	92321	92354	-	CDS(ycf2)	TCGATATTTCACGATCAAGAATGTAGTACTTTT(X2)
7	81	F	21390	23614	-	CDS(psaA)，CDS(psaB)	CTATGGCTGACCGATATTGCACATCATCATTTAGCTATTGCAGTTCTTTTTCTCATTGCCGGTCATATGTATA GAACTAAC
8	87	F	25075	25303	-	IGS(psaB-rps14)	CTTATATTTCTACATCTAGGATCCGACTTTTCTCATTGATACTAATAGGAAATGAACCATCATGGCAAAGA AAAGTTTGAT

9	250	F	71487	88489	-	IGS(rps12-clpP),IGS(rps19-rpl2)	AATCCCTCGTCGTCATAGTTGATACCTCCTCATTAAGTATAATTAATTACAATATACATGATCACAATAACAACAAAGATCACTTTGTCGACTGGTGATTTCATAGCTGTTTACTCCTTTTCTTTTAGTTTTTGATAGATGTTTTTATTGATCAGTGTCAGTACTATTACTGACACTGATCTTGAATTTTGTATATCAAATTCTATTCATTTACTCTCACACTATTTACCGCCGAACCAACGCCTATACA
10	191	F	72187	72648	-	IGS(rps12-clpP)	ATTCTTTTGAAAATAATTAATAATATGTTATACGAGACAAAGACTCACGAGTAATAACGAATAATCACTCGAATAGAAATCTCTACAAGAGCATACAGAAGTAAATCCTTGAATGTCATATAATAGTTGACCCCCTCCTTGGGTTTTGTTTACAAAATAAATAAAATAAGAAGAAAATATTCTTTTGAAAA
11	106	F	87433	88184	-	IGS(rps3-rps19)	GCAGAGCTACCATACCAATCCAAGTCAATTTGTTGAATGTGATAGTTGTTGACCTCCATTTATGTATAGCTAAACGAACAAGCCAAGTCAATTTGTTGAATGGCAT
12	91	F	91392	92668	-	IGS(rpl23-trnI-CAU),CDS(ycf2)	CCAATCGTTCTTTTTTCTCTGACCGATGGTCAGAACTTCATCTGGATTTAAATCCTACTGAGAGGTCCACTAGAAATCGGAAATCATTTAA
13	104	F	105888	106053	-	IGS(rrn16S-trnI-GAU)	AGATTTCTGTCTGGTGAAGCAACACTGAGAGCTCATGAGCTTAGTATCCTAGGACGGGACAAGATGATCAAGTGAGAGGAGCTAATTCTTGTTCGAGCTAGTAG

T: Tandem repeats;F: Forward repeats; P: palindrome repeats.position1:starting position of the first part;position2:starting position of the second part;position3:starting position of the third part.

Table S5 K2pvalues between the four *Caragana* species for IGS and introns.

	Type	C.microphylla vs C.kozlowii	C.microphylla vs C.rosea	C.kozlowii vs C.rosea	C.korshinskii vs C.microphylla	C.korshinskii vs C.kozlowii	C.korshinskii vs C.rosea
accD-psaI	IGS	0.0264	0.0389	0.0507	0	0.0264	0.0389
atpA-trnR-UCU	IGS	0.0221	0.0495	0.0633	0	0.0221	0.0495
atpB-atpE	IGS	0	0	0	0	0	0
atpE-trnM-CAU	IGS	0.0247	0.0304	0.0467	0	0.0247	0.0304
atpF-atpA	IGS	0	0.0268	0.0268	0	0	0.0268
atpH-atpF	IGS	0.0341	0.0541	0.0621	0	0.0341	0.0541
atpI-atpH	IGS	0.0288	0.0189	0.0291	0	0.0288	0.0189
ccsA-trnL-UAG	IGS	0.0085	0.0088	0.0178	0	0.0085	0.0088
cemA-petA	IGS	0.0051	0.0214	0.0262	0	0.0051	0.0214
clpP-psbB	IGS	0.0132	0.019	0.019	0	0.0132	0.019
matK-rbcL	IGS	0.0867	0.0221	0.1029	0	0.0867	0.0221
ndhA-ndhI	IGS	0	0.0126	0.0126	0	0	0.0126
ndhB-rps7	IGS	0.0027	0.0112	0.0141	0	0.0027	0.0112

ndhC-ndhK	IGS	0	0	0	0	0	0
ndhD-ccsA	IGS	0.0279	0.0443	0.04	0	0.0279	0.0443
ndhE-psaC	IGS	0.0142	0.0139	0.0214	0	0.0142	0.0139
ndhF-trnH-GUG	IGS	0.0273	0.009	0.0227	0	0.0273	0.009
ndhG-ndhE	IGS	0.0144	0.0291	0.0242	0	0.0144	0.0291
ndhH-ndhA	IGS	0	0	0	0	0	0
ndhI-ndhG	IGS	0.0391	0.0544	0.0721	0	0.0391	0.0544
ndhJ-trnF-GAA	IGS	0.0174	0.0219	0.0278	0	0.0174	0.0219
ndhK-ndhJ	IGS	0.007	0.0149	0.0074	0	0.007	0.0149
petA-psbJ	IGS	0.0242	0.0196	0.034	0	0.0242	0.0196
petB-petD	IGS	0.0149	0.03	0.0353	0	0.0149	0.03
petD-rpoA	IGS	0.0049	0.015	0.02	0	0.0049	0.015
petG-trnW-CCA	IGS	0	0.0078	0.0076	0	0	0.0078
petL-petG	IGS	0.0309	0.0388	0.0256	0	0.0309	0.0388
petN-trnC-GCA	IGS	0.0155	0.0329	0.0316	0	0.0155	0.0329
psaA-psaB	IGS	0	0	0	0	0	0

psaB-rps14	IGS	0.0165	0.0772	0.0953	0	0.0165	0.0772
psaC-ndhD	IGS	0.017	0.0077	0.017	0	0.017	0.0077
psaI-ycf4	IGS	0.017	0.0234	0.0343	0	0.017	0.0234
psaJ-rpl33	IGS	0.0209	0.019	0.0235	0	0.0209	0.019
psbA-trnK-UUU	IGS	0.0351	0.0034	0.0454	0	0.0351	0.0034
psbB-psbT	IGS	0.0202	0.0305	0.0306	0	0.0202	0.0305
psbC-psbD	IGS	0	0	0	0	0	0
psbD-trnT-GGU	IGS	0.0184	0.0234	0.0297	0	0.0184	0.0234
psbE-petL	IGS	0.0139	0.017	0.0253	0	0.0139	0.017
psbF-psbE	IGS	0.1217	0	0.1217	0	0.1217	0
psbH-petB	IGS	0.0153	0.0076	0.0231	0	0.0153	0.0076
psbI-psbK	IGS	0.0185	0.0205	0.0277	0	0.0185	0.0205
psbJ-psbL	IGS	0.0074	0.0147	0.0073	0	0.0074	0.0147
psbK-trnQ-UUG	IGS	0.0234	0.0423	0.0573	0	0.0234	0.0423
psbL-psbF	IGS	0	0.0971	0.0971	0	0	0.0971
psbM-petN	IGS	0.02	0.0308	0.0382	0	0.02	0.0308

psbN-psbH	IGS	0.046	0.0273	0.0556	0	0.046	0.0273
psbT-psbN	IGS	0	0	0	0	0	0
psbZ-trnS-UGA	IGS	0.0169	0.0167	0.0227	0	0.0172	0.017
rbcL-atpB	IGS	0.0106	0.0081	0.0164	0	0.0106	0.0081
rpl14-rpl16	IGS	0	0.017	0.017	0	0	0.017
rpl16-rps3	IGS	0.017	0.017	0.0141	0	0.017	0.017
rpl20-rps12	IGS	0.0087	0.0115	0.0205	0	0.0087	0.0115
rpl23-trnI-CAU	IGS	0.1906	0.2268	0.1673	0	0.1906	0.2268
rpl2-rpl23	IGS	0	0	0.0524	0	0	0
rpl32-ndhF	IGS	0.0328	0.029	0.0202	0	0.0328	0.029
rpl33-rps18	IGS	0.0265	0.0192	0.0309	0	0.0265	0.0192
rpl36-rps8	IGS	0.0095	0.0242	0.0242	0	0.0095	0.0242
rpoA-rps11	IGS	0.026	0.026	0.0534	0	0.026	0.026
rpoB-rpoC1	IGS	0	0	0	0	0	0
rpoC1-rpoC2	IGS	0.0105	0.0105	0.0105	0	0.0105	0.0105
rpoC2-rps2	IGS	0.0329	0.0244	0.0333	0	0.0329	0.0244

rps11-rpl36	IGS	0.0196	0.0576	0.0645	0	0.0196	0.0576
rps12-clpP	IGS	0.3094	0.3339	0.4627	0	0.3094	0.3339
rps12-trnN-GUU	IGS	0.0133	0.0162	0.0247	0	0.0133	0.0162
rps14-trnfM-CAU	IGS	0.019	0.0253	0.0189	0	0.019	0.0253
rps15-ndhH	IGS	0	0.0104	0.0104	0	0	0.0104
rps18-rpl20	IGS	0.0241	0.0393	0.0393	0	0.0241	0.0393
rps19-rpl2	IGS	0.395	0.6207	0.5641	0.0014	0.2712	0.5176
rps2-atpI	IGS	0.0397	0.0213	0.0353	0	0.0397	0.0213
rps3-rps19	IGS	0.0564	0.0917	0.1178	0	0.0596	0.0917
rps4-trnS-GGA	IGS	0.0185	0.0232	0.0328	0	0.0185	0.0232
rps7-rps12	IGS	0	0.0191	0.0191	0	0	0.0188
rps8-rpl14	IGS	0.0566	0.0485	0.0468	0	0.0566	0.0485
rrn16S-trnI-GAU	IGS	0.1259	0.1462	0.1464	0	0.1259	0.1457
rrn23S-rrn4.5S	IGS	0	0.0103	0.0103	0	0	0.0103
rrn4.5S-rrn5S	IGS	0.0185	0.009	0.0185	0	0.0185	0.009
rrn5S-trnR-ACG	IGS	0.0574	0.0309	0.031	0	0.0565	0.0309

trnA-UGC-rrn23S	IGS	0.0448	0.0289	0.0289	0	0.0448	0.0289
trnC-GCA-rpoB	IGS	0.0127	0.0184	0.0221	0	0.0127	0.0184
trnD-GUC-psbM	IGS	0.0157	0.021	0.0236	0	0.0157	0.021
trnE-UUC-trnY-GUA	IGS	0.0175	0	0.017	0	0.0175	0
trnF-GAA-trnL-UAA	IGS	0.0276	0.0246	0.0248	0	0.0276	0.0246
trnfM-CAU-trnG-GCC	IGS	0.0409	0.0348	0.0253	0	0.0409	0.0348
trnG-GCC-psbZ	IGS	0.0304	0.0336	0.0697	0	0.0304	0.0336
trnG-UCC-trnS-GCU	IGS	-	-	-	0	-	0.0175
trnH-GUG-psbA	IGS	0.0315	0.0327	0.056	0	0.0315	0.0327
trnI-CAU-ycf2	IGS	0.1925	0.2366	0.3169	0.0301	0.1693	0.2142
trnI-GAU-trnA-UGC	IGS	0.0323	0.0484	0.0492	0	0.0323	0.0484
trnK-UUU-matK	IGS	0.0093	0.0142	0.0174	0	0.0093	0.0142
trnL-CAA-ndhB	IGS	0.0094	0.0192	0.0202	0	0.0094	0.0192
trnL-UAA-trnT-UGU	IGS	0.0347	0.0319	0.0267	0.0006	0.0353	0.0319
trnL-UAG-rpl32	IGS	0.0268	0.025	0.0322	0	0.0268	0.025
trnM-CAU-trnV-UAC	IGS	0.0297	0.0297	0.0353	0	0.0297	0.0297

trnN-GUU-trnV-GAC	IGS	0.0154	0.0155	0.0314	0	0.0154	0.0155
trnN-GUU-ycf1	IGS	0.0546	0.1366	0.1579	0.068	0.0892	0.0726
trnP-UGG-psaJ	IGS	0.0222	0.0377	0.0397	0	0.0222	0.0377
trnQ-UUG-accD	IGS	0.041	0.0203	0.0463	0	0.041	0.0203
trnR-ACG-trnN-GUU	IGS	0.0277	0.0412	0.0263	0	0.0277	0.0412
trnR-UCU-trnG-UCC	IGS	0.026	0.0499	0.0366	0	0.026	0.0499
trnS-GCU-psbI	IGS	0	0.0111	0.0113	0	0	0.0111
trnS-GGA-ycf3	IGS	0	0.0112	0.0114	0	0	0.0113
trnS-UGA-psbC	IGS	0.0094	0.053	0.0431	0	0.0094	0.053
trnT-GGU-trnE-UUC	IGS	0.0423	0.0243	0.0329	0	0.0423	0.0243
trnT-UGU-rps4	IGS	0.011	0.015	0.0192	0	0.011	0.015
trnV-GAC-rrn16S	IGS	0.0045	0.0513	0.0494	0	0.0045	0.0513
trnV-UAC-ndhC	IGS	0.0309	0.0484	0.0703	0	0.0309	0.0484
trnW-CCA-trnP-UGG	IGS	0	0.0247	0.0275	0	0	0.0247
trnY-GUA-trnD-GUC	IGS	0.0142	0.0209	0.0163	0	0.0142	0.0209
ycf1-rps15	IGS	0.0198	0.4627	0.4782	0	0.0198	0.4627

ycf2-trnL-CAA	IGS	0.003	0.003	0.0061	0	0.003	0.003
ycf3-psaA	IGS	0.0196	0.0207	0.0355	0.0059	0.0134	0.027
ycf4-cemA	IGS	0.0313	0.0085	0.0377	0	0.0313	0.0085
ycf3-1	intron	0.0028	0.0058	0.0087	0	0.0028	0.0058
trnG-UCC	intron	0	0.0164	0	0	0	0.0164
trnL-CAA	intron	0.0092	0.0039	0.0077	0	0.0092	0.0039
ndhB	intron	0.0059	0.0089	0.0105	0	0.0059	0.0089
atpF	intron	0.0076	0.0061	0.0137	0	0.0076	0.0061
petD	intron	0.007	0.0114	0.0071	0	0.007	0.0114
rpoC1	intron	0.0051	0.0115	0.0116	0	0.0051	0.0115
trnV-UAC	intron	0.0053	0.0141	0.0123	0	0.0053	0.0141
petB	intron	0.0062	0.0148	0.0136	0	0.0062	0.0148
trnI-AUC	intron	0.0127	0.0118	0.0128	0	0.0127	0.0118
trnK-UUU	intron	0.0101	0.0138	0.0171	0	0.0101	0.0138
rpl2	intron	0.0163	0.0124	0.0077	0	0.0163	0.0124
trnA-UGC	intron	0.0125	0.0125	0.0163	0	0.0125	0.0125

rpl16	intron	0.0119	0.0156	0.0163	0	0.0119	0.0156
ndhA	intron	0.02	0.0182	0.0269	0	0.02	0.0182
clpP	intron	0.0251	0.0179	0.0214	0.0015	0.0259	0.0183
ycf3-2	intron	0.0259	0.0257	0.0236	0	0.0259	0.0257

Table S6 The PCR primers used in this study.

Primer ID	Sequence (5′-3′)	Primer ID	Sequence (5′-3′)
A_1_F	CCTTACCATGGCGTTACTCTAC	D_1_F	ACCAAACAGAGCTACCATACC
A_1_R	GGAAGAGGCGAGGCATTAAA	D_1_R	CTTTCCCTCGTTACCCTCATT
A_2_F	CCTGAATAAGGAACCAGCCAATA	D_2_F	GTGATAGTTGTTGACCTCCATTT
A_2_R	CTATCTCACATTAAGCCGGGAAA	D_2_R	GGTTGTTATTTCCTCCTTTCCC
B_1_F	GGGATAGGGATGACAGGATTTG	E_1_F	GAGGTCCACTAGAAATCGGAAA
B_1_R	CTGGTGCCACAGAGAGATATG	E_1_R	CCTGTGAATTGCGTGAAAGTAG
B_2_F	TAGTTCGGGATAGGGATGACA	E_2_F	TTTAAATCCTACTGAGAGGTCCA
B_2_R	GTGGAAGTCCGAATTCAATCAAA	E_2_R	GTGAATGAAAGATACCGCTGAA
C_1_F	AGTGTATCGTGATGGGTTTGTT	F_1_F	ATTGCGGTCAAGTTTTGGCT
C_1_R	GGGTAATATCCGGAATGGCTATG	F_1_R	CCCAGTCCATTTGTTGAATGTGA
C_2_F	TTTCGTTCTCATGATAGTGTATCG	F_2_F	ATAATTGCGGTCAAGTTTTGGCT
C_2_R	TGTAGAAGCAGAAGTACAGACA	F_2_R	GCTAGAATACAAATCCCAGTCCA

The first two letters in each primer's ID represent the names of the adjacent scaffolds, and the gap between the scaffolds was filled by this primer. F and R represent "Forward" and "Reverse," respectively.

Table S7 List of chloroplast genome sequences used in this study.

#Organism/Na me	Grou p	SubGr oup	Type	RefSeq	INSDC	Size (Kb)	GC%	Prote in	rRN A	tRN A	Ge ne	Pseudo-gene	Release Date	Modify Date
Astragalus mongholicus	Plants	Land Plants	chloroplast	NC_029 828.1	KU6665 54	123.5 82	34.09 07	76	4	28	108	-	04-09-20 16	04-09-20 16
Astragalus mongholicus var. *nakaianus*	Plants	Land Plants	chloroplast	NC_028 171.1	KR2967 89	123.6 33	34.07 26	75	4	30	110	1	11-01-20 15	11-01-20 15
Caragana korshinskii	Plants	Land Plants	chloroplast	NC_035 229.1	KX2899 23	129.3 31	0	76	4	31	111	-	07-14-20 17	07-14-20 17
Caragana kozlowii	Plants	Land Plants	chloroplast	NC_035 228.1	KX3492 19	131.2 74	34.49 5	76	4	30	110	-	07-14-20 17	07-14-20 17
Caragana microphylla	Plants	Land Plants	chloroplast	NC_032 691.1	KX2899 22	130.0 29	34.26 24	76	4	31	111	-	01-05-20 17	01-05-20 17
Cicer arietinum	Plants	Land Plants	chloroplast	NC_011 163.1	EU8358 53	125.3 19	33.90 63	75	4	29	108	-	08-26-20 08	03-26-20 10
Glycyrrhiza glabra	Plants	Land Plants	chloroplast	NC_024 038.1	KF20159 0	127.9 43	34.23 79	76	4	30	110	-	05-01-20 14	09-29-20 14
Glycyrrhiza	Plants	Land	chloroplast	NC_034	KY0384	127.9	34.24	76	4	30	110	-	03-24-20	03-24-20

lepidota		Plants		229.1	82	39	21						17	17
Lathyrus clymenum	Plants	Land Plants	chloroplast	NC_027 148.1	KJ85023 5	121.2 63	34.94 88	75	4	31	110	-	06-02-20 15	06-03-20 15
Lathyrus davidii	Plants	Land Plants	chloroplast	NC_027 073.1	KJ80619 2	123.8 95	34.88 76	74	4	30	108	-	05-14-20 15	05-14-20 15
Lathyrus graminifolius	Plants	Land Plants	chloroplast	NC_027 074.1	KJ80619 3	122.4 38	34.95 89	75	4	30	109	-	05-14-20 15	05-14-20 15
Lathyrus inconspicuus	Plants	Land Plants	chloroplast	NC_027 149.1	KJ85023 6	123.1 53	34.74 46	74	4	30	108	-	06-02-20 15	06-03-20 15
Lathyrus japonicus	Plants	Land Plants	chloroplast	NC_027 075.1	KJ80619 4	124.2 42	34.90 2	75	4	30	109	-	05-14-20 15	05-14-20 15
Lathyrus littoralis	Plants	Land Plants	chloroplast	NC_027 076.1	KJ80619 6	123.7 34	34.81 5	74	4	30	108	-	05-14-20 15	05-14-20 15
Lathyrus ochroleucus	Plants	Land Plants	chloroplast	NC_027 077.1	KJ80619 7	123.9 11	34.91 86	75	4	30	109	-	05-14-20 15	05-14-20 15
Lathyrus odoratus	Plants	Land Plants	chloroplast	NC_027 150.1	KJ85023 7	120.2 89	35.15 95	73	4	30	107	-	06-02-20 15	06-03-20 15
Lathyrus palustris	Plants	Land Plants	chloroplast	NC_027 078.1	KJ80619 9	124.2 87	34.85 24	75	4	30	109	-	05-14-20 15	05-14-20 15

Lathyrus pubescens	Plants	Land Plants	chloroplast	NC_027079.1	KJ806200	126.421	35.0061	74	4	30	108	-	05-14-2015	05-14-2015
Lathyrus sativus	Plants	Land Plants	chloroplast	NC_014063.1	HM029371	121.02	35.1124	74	4	30	109	1	04-23-2010	12-07-2010
Lathyrus tingitanus	Plants	Land Plants	chloroplast	NC_027151.1	KJ850238	122.165	34.8741	74	4	30	108	-	06-02-2015	06-03-2015
Lathyrus venosus	Plants	Land Plants	chloroplast	NC_027080.1	KJ806202	125.459	34.8759	75	5	30	110	-	05-14-2015	05-14-2015
Lens culinaris	Plants	Land Plants	chloroplast	NC_027152.1	KJ850239	122.967	34.4255	73	4	30	108	1	06-02-2015	06-03-2015
Medicago falcata 1210	Plants	Land Plants	chloroplast	NC_032066.1	KX831887	124.43	33.9572	71	4	24	101	2	12-07-2016	12-07-2016
Medicago hybrida	Plants	Land Plants	chloroplast	NC_027153.1	KJ850240	125.208	33.8173	75	4	30	109	-	06-02-2015	06-03-2015
Medicago papillosa	Plants	Land Plants	chloroplast	NC_027154.1	KJ850241	125.203	33.8307	75	4	30	109	-	06-02-2015	06-03-2015
Medicago truncatula Variety Jema Long A-17	Plants	Land Plants	chloroplast	NC_003119.6	AC093544	124.033	33.9724	76	4	29	109	-	08-31-2001	04-15-2009

Pisum sativum	Plants	Land Plants	chloroplast	NC_014057.1	HM029370	122.169	34.828	74	4	30	110	2	04-23-2010	12-07-2010
Trifolium boissieri	Plants	Land Plants	chloroplast	NC_025743.1	KJ788284	125.74	34.8091	74	5	31	110	-	01-13-2015	01-13-2015
Trifolium glanduliferum	Plants	Land Plants	chloroplast	NC_025744.1	KJ788285	126.149	34.4632	75	5	30	110	-	12-03-2014	12-03-2014
Trifolium strictum	Plants	Land Plants	chloroplast	NC_025745.1	KJ788292	125.834	34.5392	75	5	31	111	-	01-29-2015	01-29-2015
Vicia sativa	Plants	Land Plants	chloroplast	NC_027155.1	KJ850242	122.467	35.1507	75	4	29	109	1	06-02-2015	06-03-2015
Wisteria floribunda	Plants	Land Plants	chloroplast	NC_027677.1	KM103376	130.96	34.3288	76	4	30	110	-	08-04-2015	11-17-2015
Wisteria sinensis	Plants	Land Plants	chloroplast	NC_029406.1	KT200359	130.561	34.3962	75	4	30	109	-	03-01-2016	03-01-2016
Arabidopsis thaliana	Plants	Land Plants	chloroplast	NC_000932.1	AP000423	154.478	36.29	85	7	37	129	-	09-15-1999	03-26-2010
Nicotiana tabacum	Plants	Land Plants	plastid	NC_001879.2	Z00044	155.943	37.85	98	8	37	144	1	11-18-1986	04-15-2009
